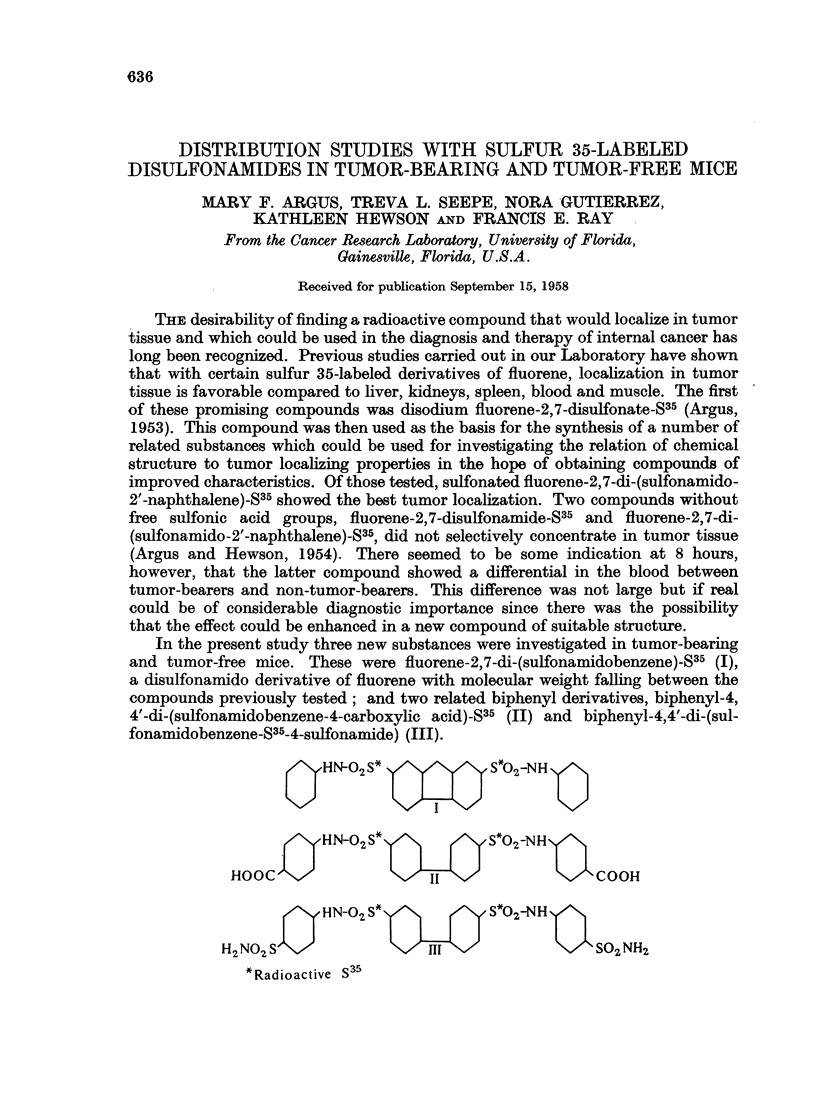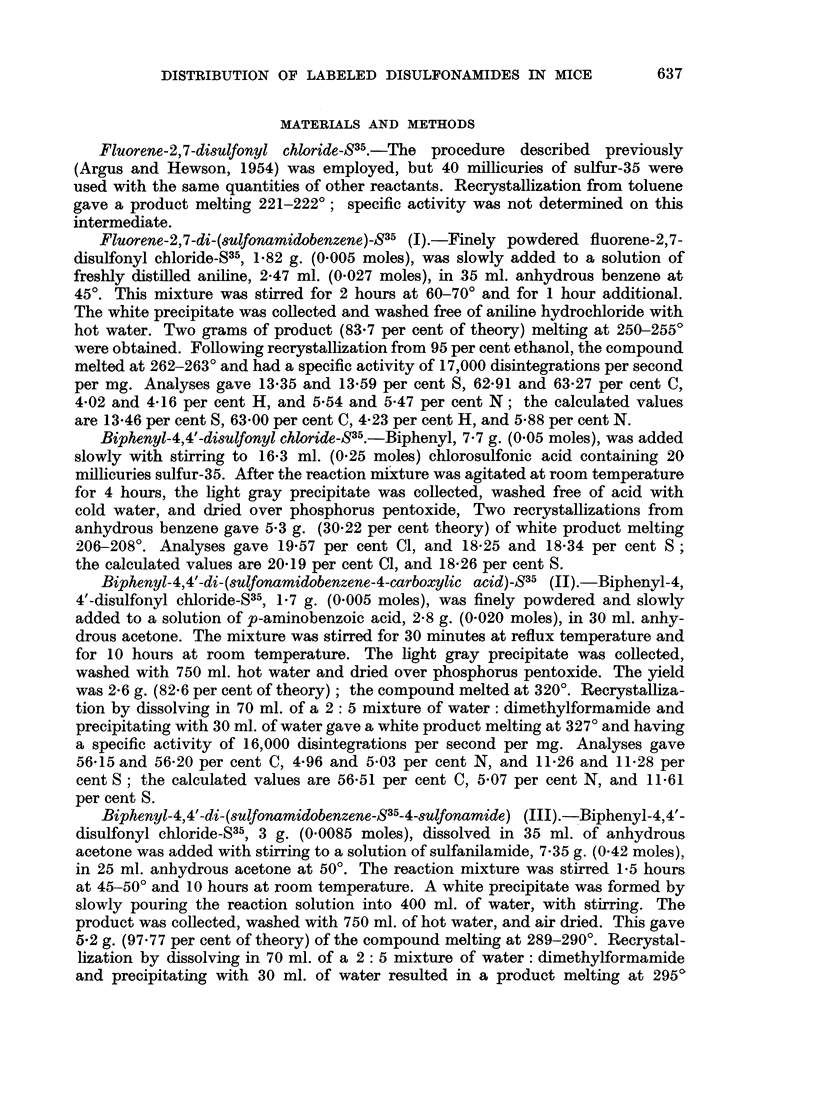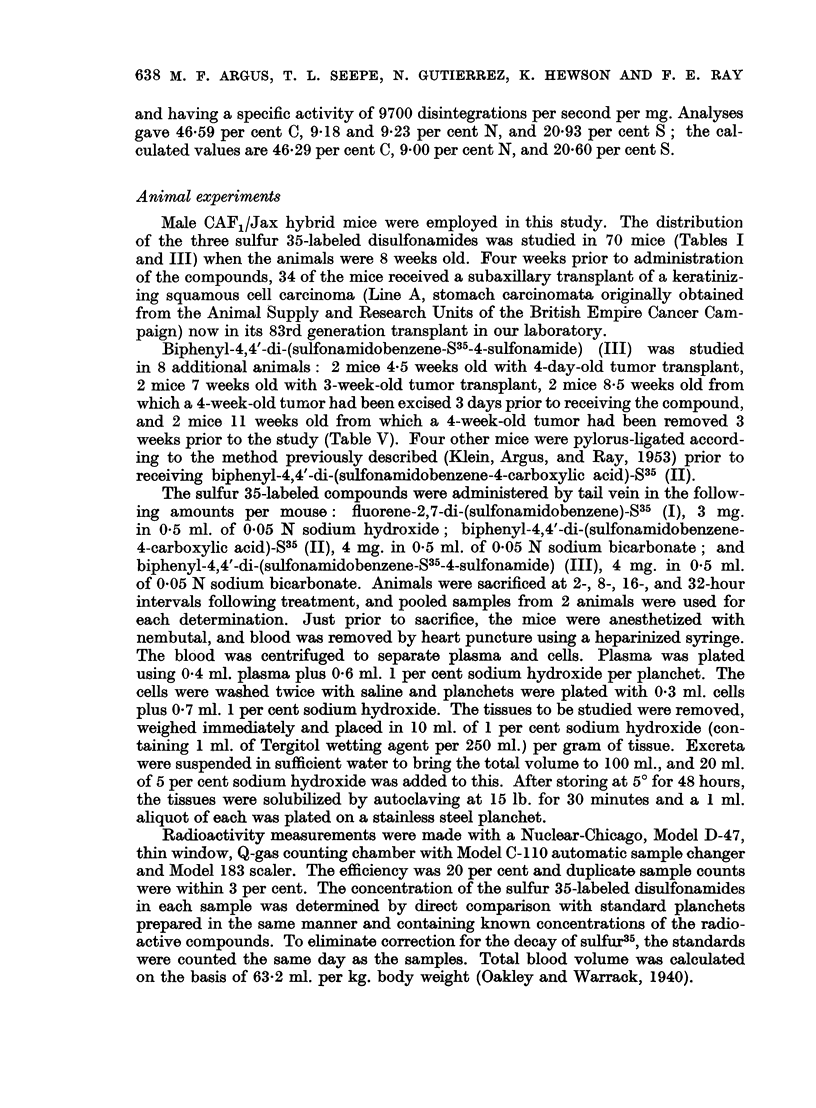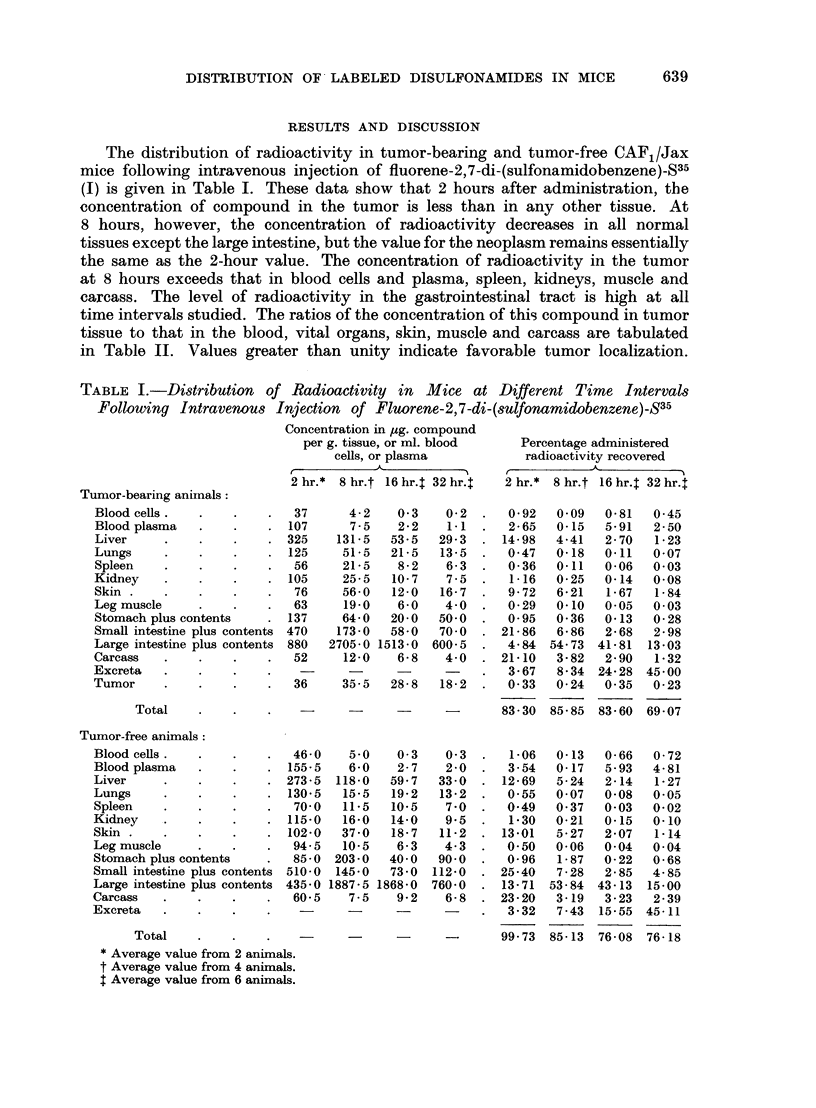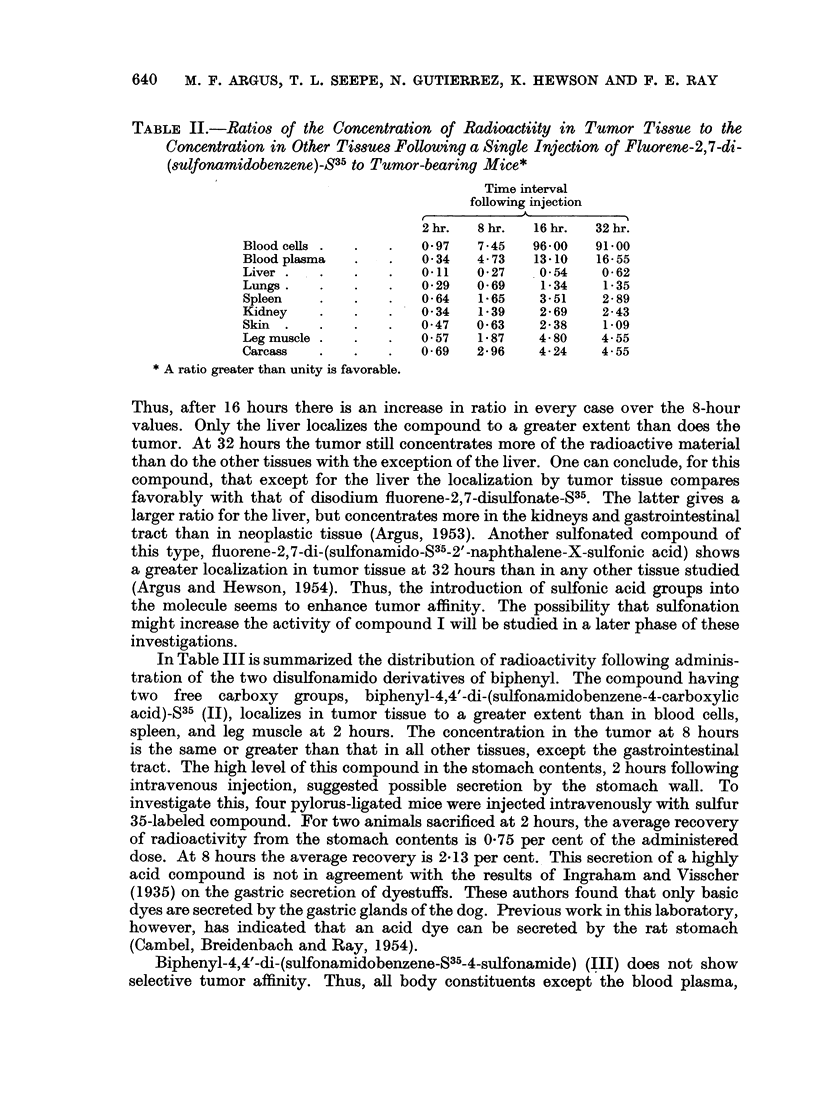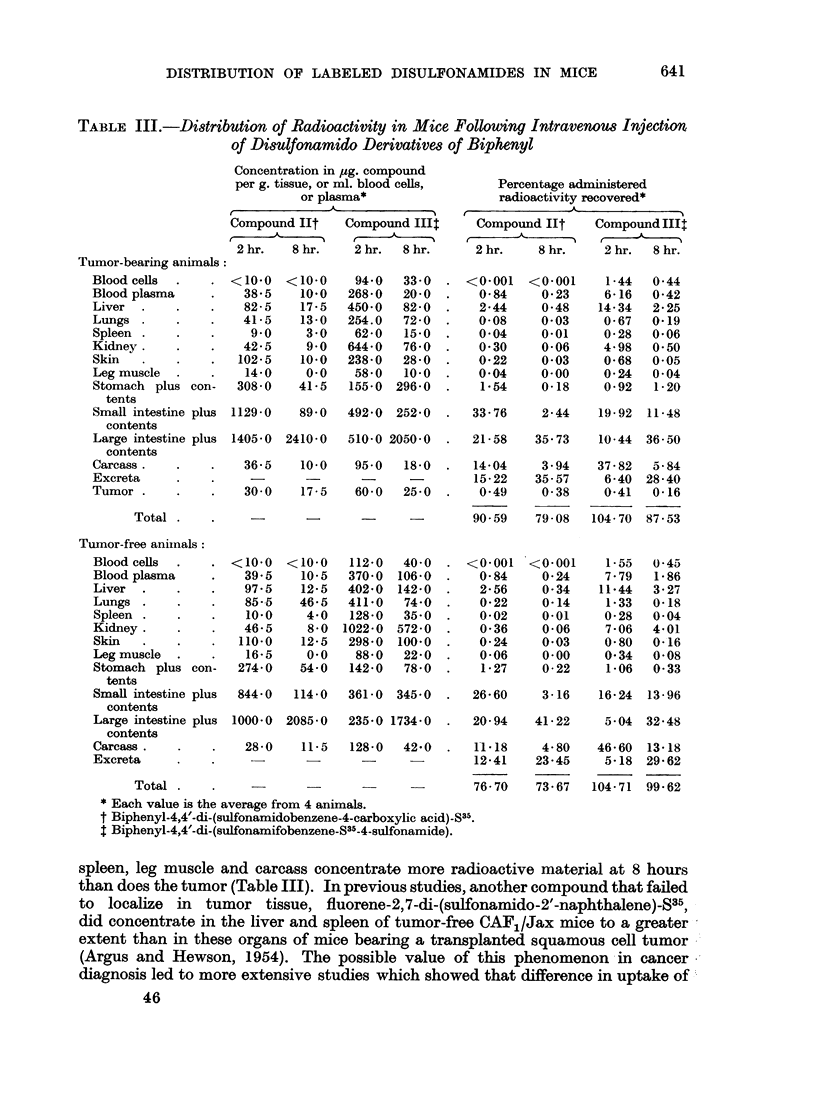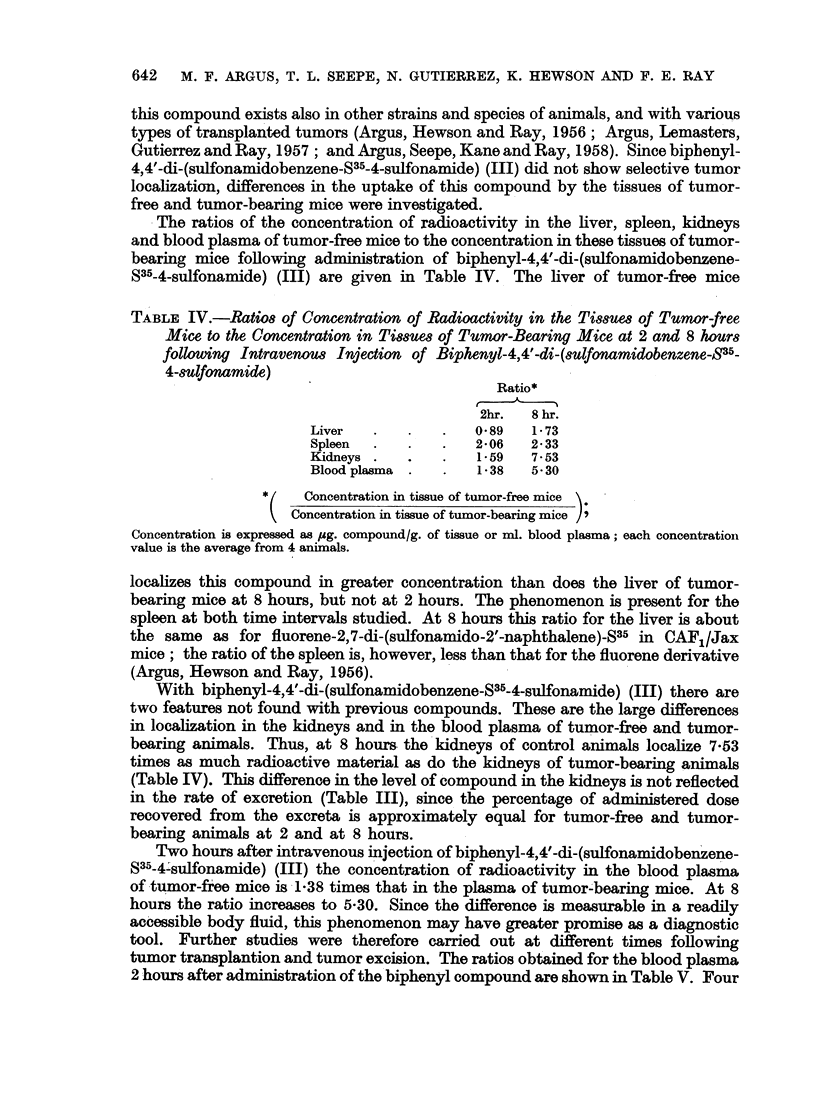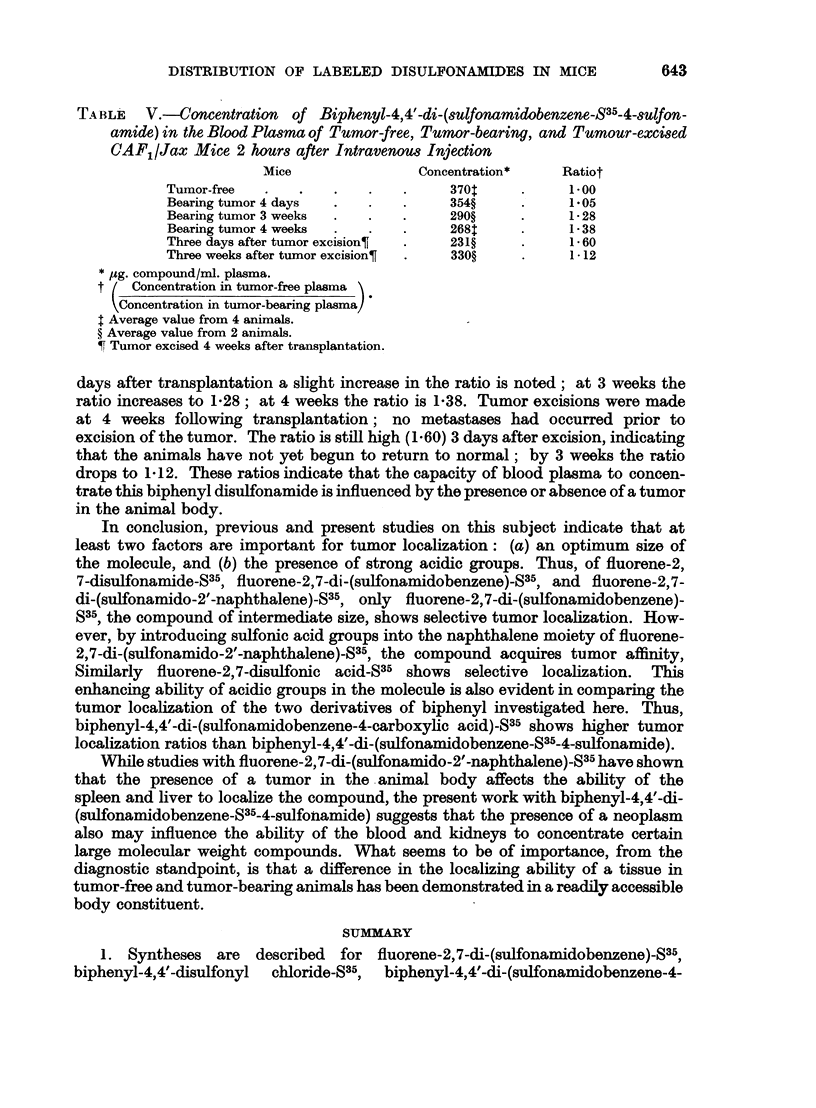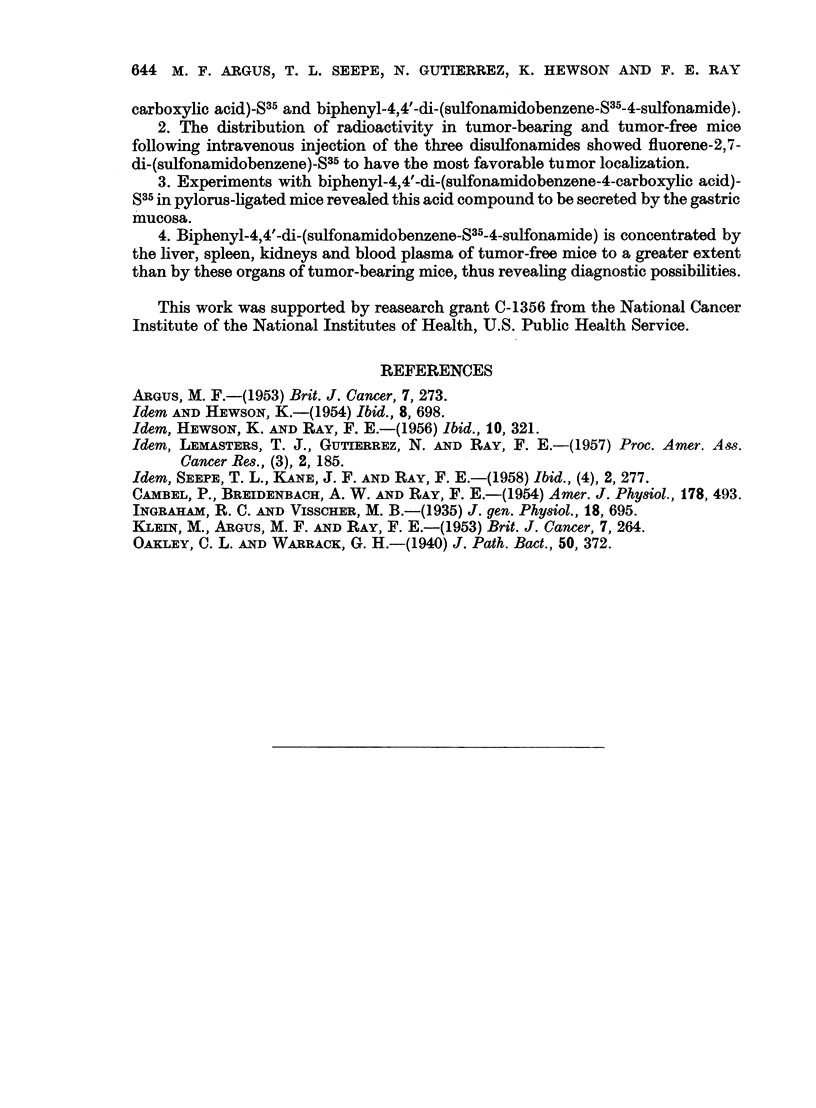# Distribution Studies with Sulfur 35-Labeled Disulfonamides in Tumor-bearing and Tumor-free Mice

**DOI:** 10.1038/bjc.1958.72

**Published:** 1958-12

**Authors:** Mary F. Argus, Treva L. Seepe, Nora Gutierrez, Kathleen Hewson, Francis E. Ray


### DISTRIBUTION STUDIES WITH SULFUR 35-LABELED DISULFONAMIDES IN TUMOR-BEARING AND TUMOR-FREE MICE

MARY F. ARGUS, TREVA L. SEEPE, NORA GUTIERREZ, KATHLEEN HEWSON AND FRANCIS E. RAY

*From the Cancer Research Laboratory, University of Florida, Gainesville, Florida, U.S.A.*

Received for publication September 15, 1958

THE desirability of finding a radioactive compound that would localize in tumor tissue and which could be used in the diagnosis and therapy of internal cancer has long been recognized. Previous studies carried out in our Laboratory have shown that with certain sulfur 35-labeled derivatives of fluorene, localization in tumor tissue is favorable compared to liver, kidneys, spleen, blood and muscle. The first of these promising compounds was disodium fluorene-2,7-disulfonate-$S^{35}$ (Argus, 1953). This compound was then used as the basis for the synthesis of a number of related substances which could be used for investigating the relation of chemical structure to tumor localizing properties in the hope of obtaining compounds of improved characteristics. Of those tested, sulfonated fluorene-2,7-di-(sulfonamido-2′-naphthalene)-$S^{35}$ showed the best tumor localization. Two compounds without free sulfonic acid groups, fluorene-2,7-disulfonamide-$S^{35}$ and fluorene-2,7-di-(sulfonamido-2′-naphthalene)-$S^{35}$, did not selectively concentrate in tumor tissue (Argus and Hewson, 1954). There seemed to be some indication at 8 hours, however, that the latter compound showed a differential in the blood between tumor-bearers and non-tumor-bearers. This difference was not large but if real could be of considerable diagnostic importance since there was the possibility that the effect could be enhanced in a new compound of suitable structure.

In the present study three new substances were investigated in tumor-bearing and tumor-free mice. These were fluorene-2,7-di-(sulfonamidobenzene)-$S^{35}$ (I), a disulfonamido derivative of fluorene with molecular weight falling between the compounds previously tested ; and two related biphenyl derivatives, biphenyl-4,4′-di-(sulfonamidobenzene-4-carboxylic acid)-$S^{35}$ (II) and biphenyl-4,4′-di-(sulfonamidobenzene-$S^{35}$-4-sulfonamide) (III).

*Radioactive $S^{35}$

### MATERIALS AND METHODS

*Fluorene-2,7-disulfonyl chloride-$S^{35}$*.—The procedure described previously (Argus and Hewson, 1954) was employed, but 40 millicuries of sulfur-35 were used with the same quantities of other reactants. Recrystallization from toluene gave a product melting 221–222° ; specific activity was not determined on this intermediate.

*Fluorene-2,7-di-(sulfonamidobenzene)-$S^{35}$* (I).—Finely powdered fluorene-2,7-disulfonyl chloride-$S^{35}$, 1·82 g. (0·005 moles), was slowly added to a solution of freshly distilled aniline, 2·47 ml. (0·027 moles), in 35 ml. anhydrous benzene at 45°. This mixture was stirred for 2 hours at 60–70° and for 1 hour additional. The white precipitate was collected and washed free of aniline hydrochloride with hot water. Two grams of product (83·7 per cent of theory) melting at 250–255° were obtained. Following recrystallization from 95 per cent ethanol, the compound melted at 262–263° and had a specific activity of 17,000 disintegrations per second per mg. Analyses gave 13·35 and 13·59 per cent S, 62·91 and 63·27 per cent C, 4·02 and 4·16 per cent H, and 5·54 and 5·47 per cent N ; the calculated values are 13·46 per cent S, 63·00 per cent C, 4·23 per cent H, and 5·88 per cent N.

*Biphenyl-4,4′-disulfonyl chloride-$S^{35}$*.—Biphenyl, 7·7 g. (0·05 moles), was added slowly with stirring to 16·3 ml. (0·25 moles) chlorosulfonic acid containing 20 millicuries sulfur-35. After the reaction mixture was agitated at room temperature for 4 hours, the light gray precipitate was collected, washed free of acid with cold water, and dried over phosphorus pentoxide, Two recrystallizations from anhydrous benzene gave 5·3 g. (30·22 per cent theory) of white product melting 206–208°. Analyses gave 19·57 per cent Cl, and 18·25 and 18·34 per cent S ; the calculated values are 20·19 per cent Cl, and 18·26 per cent S.

*Biphenyl-4,4′-di-(sulfonamidobenzene-4-carboxylic acid)-$S^{35}$* (II).—Biphenyl-4,4′-disulfonyl chloride-$S^{35}$, 1·7 g. (0·005 moles), was finely powdered and slowly added to a solution of *p*-aminobenzoic acid, 2·8 g. (0·020 moles), in 30 ml. anhydrous acetone. The mixture was stirred for 30 minutes at reflux temperature and for 10 hours at room temperature. The light gray precipitate was collected, washed with 750 ml. hot water and dried over phosphorus pentoxide. The yield was 2·6 g. (82·6 per cent of theory) ; the compound melted at 320°. Recrystallization by dissolving in 70 ml. of a 2 : 5 mixture of water : dimethylformamide and precipitating with 30 ml. of water gave a white product melting at 327° and having a specific activity of 16,000 disintegrations per second per mg. Analyses gave 56·15 and 56·20 per cent C, 4·96 and 5·03 per cent N, and 11·26 and 11·28 per cent S ; the calculated values are 56·51 per cent C, 5·07 per cent N, and 11·61 per cent S.

*Biphenyl-4,4′-di-(sulfonamidobenzene-$S^{35}$-4-sulfonamide)* (III).—Biphenyl-4,4′-disulfonyl chloride-$S^{35}$, 3 g. (0·0085 moles), dissolved in 35 ml. of anhydrous acetone was added with stirring to a solution of sulfanilamide, 7·35 g. (0·42 moles), in 25 ml. anhydrous acetone at 50°. The reaction mixture was stirred 1·5 hours at 45–50° and 10 hours at room temperature. A white precipitate was formed by slowly pouring the reaction solution into 400 ml. of water, with stirring. The product was collected, washed with 750 ml. of hot water, and air dried. This gave 5·2 g. (97·77 per cent of theory) of the compound melting at 289–290°. Recrystallization by dissolving in 70 ml. of a 2 : 5 mixture of water : dimethylformamide and precipitating with 30 ml. of water resulted in a product melting at 295°

and having a specific activity of 9700 disintegrations per second per mg. Analyses gave 46·59 per cent C, 9·18 and 9·23 per cent N, and 20·93 per cent S ; the calculated values are 46·29 per cent C, 9·00 per cent N, and 20·60 per cent S.

*Animal experiments*

Male $CAF_1$/Jax hybrid mice were employed in this study. The distribution of the three sulfur 35-labeled disulfonamides was studied in 70 mice (Tables I and III) when the animals were 8 weeks old. Four weeks prior to administration of the compounds, 34 of the mice received a subaxillary transplant of a keratinizing squamous cell carcinoma (Line A, stomach carcinomata originally obtained from the Animal Supply and Research Units of the British Empire Cancer Campaign) now in its 83rd generation transplant in our laboratory.

Biphenyl-4,4′-di-(sulfonamidobenzene-$S^{35}$-4-sulfonamide) (III) was studied in 8 additional animals : 2 mice 4·5 weeks old with 4-day-old tumor transplant, 2 mice 7 weeks old with 3-week-old tumor transplant, 2 mice 8·5 weeks old from which a 4-week-old tumor had been excised 3 days prior to receiving the compound, and 2 mice 11 weeks old from which a 4-week-old tumor had been removed 3 weeks prior to the study (Table V). Four other mice were pylorus-ligated according to the method previously described (Klein, Argus, and Ray, 1953) prior to receiving biphenyl-4,4′-di-(sulfonamidobenzene-4-carboxylic acid)-$S^{35}$ (II).

The sulfur 35-labeled compounds were administered by tail vein in the following amounts per mouse : fluorene-2,7-di-(sulfonamidobenzene)-$S^{35}$ (I), 3 mg. in 0·5 ml. of 0·05 N sodium hydroxide ; biphenyl-4,4′-di-(sulfonamidobenzene-4-carboxylic acid)-$S^{35}$ (II), 4 mg. in 0·5 ml. of 0·05 N sodium bicarbonate ; and biphenyl-4,4′-di-(sulfonamidobenzene-$S^{35}$-4-sulfonamide) (III), 4 mg. in 0·5 ml. of 0·05 N sodium bicarbonate. Animals were sacrificed at 2-, 8-, 16-, and 32-hour intervals following treatment, and pooled samples from 2 animals were used for each determination. Just prior to sacrifice, the mice were anesthetized with nembutal, and blood was removed by heart puncture using a heparinized syringe. The blood was centrifuged to separate plasma and cells. Plasma was plated using 0·4 ml. plasma plus 0·6 ml. 1 per cent sodium hydroxide per planchet. The cells were washed twice with saline and planchets were plated with 0·3 ml. cells plus 0·7 ml. 1 per cent sodium hydroxide. The tissues to be studied were removed, weighed immediately and placed in 10 ml. of 1 per cent sodium hydroxide (containing 1 ml. of Tergitol wetting agent per 250 ml.) per gram of tissue. Excreta were suspended in sufficient water to bring the total volume to 100 ml., and 20 ml. of 5 per cent sodium hydroxide was added to this. After storing at 5° for 48 hours, the tissues were solubilized by autoclaving at 15 lb. for 30 minutes and a 1 ml. aliquot of each was plated on a stainless steel planchet.

Radioactivity measurements were made with a Nuclear-Chicago, Model D-47, thin window, Q-gas counting chamber with Model C-110 automatic sample changer and Model 183 scaler. The efficiency was 20 per cent and duplicate sample counts were within 3 per cent. The concentration of the sulfur 35-labeled disulfonamides in each sample was determined by direct comparison with standard planchets prepared in the same manner and containing known concentrations of the radioactive compounds. To eliminate correction for the decay of $sulfur^{35}$, the standards were counted the same day as the samples. Total blood volume was calculated on the basis of 63·2 ml. per kg. body weight (Oakley and Warrack, 1940).

### RESULTS AND DISCUSSION

The distribution of radioactivity in tumor-bearing and tumor-free $CAF_1$/Jax mice following intravenous injection of fluorene-2,7-di-(sulfonamidobenzene)-$S^{35}$ (I) is given in Table I. These data show that 2 hours after administration, the concentration of compound in the tumor is less than in any other tissue. At 8 hours, however, the concentration of radioactivity decreases in all normal tissues except the large intestine, but the value for the neoplasm remains essentially the same as the 2-hour value. The concentration of radioactivity in the tumor at 8 hours exceeds that in blood cells and plasma, spleen, kidneys, muscle and carcass. The level of radioactivity in the gastrointestinal tract is high at all time intervals studied. The ratios of the concentration of this compound in tumor tissue to that in the blood, vital organs, skin, muscle and carcass are tabulated in Table II. Values greater than unity indicate favorable tumor localization.

TABLE I.—*Distribution of Radioactivity in Mice at Different Time Intervals Following Intravenous Injection of Fluorene-2,7-di-(sulfonamidobenzene)-$S^{35}$*

| | Concentration in µg. compound per g. tissue, or ml. blood cells, or plasma | | | | Percentage administered radioactivity recovered | | | |
|---|---|---|---|---|---|---|---|---|
| | 2 hr.* | 8 hr.† | 16 hr.‡ | 32 hr.‡ | 2 hr.* | 8 hr.† | 16 hr.‡ | 32 hr.‡ |
| Tumor-bearing animals : | | | | | | | | |
| Blood cells | 37 | 4·2 | 0·3 | 0·2 | 0·92 | 0·09 | 0·81 | 0·45 |
| Blood plasma | 107 | 7·5 | 2·2 | 1·1 | 2·65 | 0·15 | 5·91 | 2·50 |
| Liver | 325 | 131·5 | 53·5 | 29·3 | 14·98 | 4·41 | 2·70 | 1·23 |
| Lungs | 125 | 51·5 | 21·5 | 13·5 | 0·47 | 0·18 | 0·11 | 0·07 |
| Spleen | 56 | 21·5 | 8·2 | 6·3 | 0·36 | 0·11 | 0·06 | 0·03 |
| Kidney | 105 | 25·5 | 10·7 | 7·5 | 1·16 | 0·25 | 0·14 | 0·08 |
| Skin | 76 | 56·0 | 12·0 | 16·7 | 9·72 | 6·21 | 1·67 | 1·84 |
| Leg muscle | 63 | 19·0 | 6·0 | 4·0 | 0·29 | 0·10 | 0·05 | 0·03 |
| Stomach plus contents | 137 | 64·0 | 20·0 | 50·0 | 0·95 | 0·36 | 0·13 | 0·28 |
| Small intestine plus contents | 470 | 173·0 | 58·0 | 70·0 | 21·86 | 6·86 | 2·68 | 2·98 |
| Large intestine plus contents | 880 | 2705·0 | 1513·0 | 600·5 | 4·84 | 54·73 | 41·81 | 13·03 |
| Carcass | 52 | 12·0 | 6·8 | 4·0 | 21·10 | 3·82 | 2·90 | 1·32 |
| Excreta | — | — | — | — | 3·67 | 8·34 | 24·28 | 45·00 |
| Tumor | 36 | 35·5 | 28·8 | 18·2 | 0·33 | 0·24 | 0·35 | 0·23 |
| Total | — | — | — | — | 83·30 | 85·85 | 83·60 | 69·07 |
| Tumor-free animals : | | | | | | | | |
| Blood cells | 46·0 | 5·0 | 0·3 | 0·3 | 1·06 | 0·13 | 0·66 | 0·72 |
| Blood plasma | 155·5 | 6·0 | 2·7 | 2·0 | 3·54 | 0·17 | 5·93 | 4·81 |
| Liver | 273·5 | 118·0 | 59·7 | 33·0 | 12·69 | 5·24 | 2·14 | 1·27 |
| Lungs | 130·5 | 15·5 | 19·2 | 13·2 | 0·55 | 0·07 | 0·08 | 0·05 |
| Spleen | 70·0 | 11·5 | 10·5 | 7·0 | 0·49 | 0·37 | 0·03 | 0·02 |
| Kidney | 115·0 | 16·0 | 14·0 | 9·5 | 1·30 | 0·21 | 0·15 | 0·10 |
| Skin | 102·0 | 37·0 | 18·7 | 11·2 | 13·01 | 5·27 | 2·07 | 1·14 |
| Leg muscle | 94·5 | 10·5 | 6·3 | 4·3 | 0·50 | 0·06 | 0·04 | 0·04 |
| Stomach plus contents | 85·0 | 203·0 | 40·0 | 90·0 | 0·96 | 1·87 | 0·22 | 0·68 |
| Small intestine plus contents | 510·0 | 145·0 | 73·0 | 112·0 | 25·40 | 7·28 | 2·85 | 4·85 |
| Large intestine plus contents | 435·0 | 1887·5 | 1868·0 | 760·0 | 13·71 | 53·84 | 43·13 | 15·00 |
| Carcass | 60·5 | 7·5 | 9·2 | 6·8 | 23·20 | 3·19 | 3·23 | 2·39 |
| Excreta | — | — | — | — | 3·32 | 7·43 | 15·55 | 45·11 |
| Total | — | — | — | — | 99·73 | 85·13 | 76·08 | 76·18 |

* Average value from 2 animals.
† Average value from 4 animals.
‡ Average value from 6 animals.

TABLE II.—*Ratios of the Concentration of Radioactiity in Tumor Tissue to the Concentration in Other Tissues Following a Single Injection of Fluorene-2,7-di-(sulfonamidobenzene)-$S^{35}$ to Tumor-bearing Mice**

| | Time interval following injection | | | |
|---|---|---|---|---|
| | 2 hr. | 8 hr. | 16 hr. | 32 hr. |
| Blood cells . . . | 0·97 | 7·45 | 96·00 | 91·00 |
| Blood plasma . . | 0·34 | 4·73 | 13·10 | 16·55 |
| Liver . . . . | 0·11 | 0·27 | 0·54 | 0·62 |
| Lungs . . . . | 0·29 | 0·69 | 1·34 | 1·35 |
| Spleen . . . | 0·64 | 1·65 | 3·51 | 2·89 |
| Kidney . . . | 0·34 | 1·39 | 2·69 | 2·43 |
| Skin . . . . | 0·47 | 0·63 | 2·38 | 1·09 |
| Leg muscle . . . | 0·57 | 1·87 | 4·80 | 4·55 |
| Carcass . . . | 0·69 | 2·96 | 4·24 | 4·55 |

* A ratio greater than unity is favorable.

Thus, after 16 hours there is an increase in ratio in every case over the 8-hour values. Only the liver localizes the compound to a greater extent than does the tumor. At 32 hours the tumor still concentrates more of the radioactive material than do the other tissues with the exception of the liver. One can conclude, for this compound, that except for the liver the localization by tumor tissue compares favorably with that of disodium fluorene-2,7-disulfonate-$S^{35}$. The latter gives a larger ratio for the liver, but concentrates more in the kidneys and gastrointestinal tract than in neoplastic tissue (Argus, 1953). Another sulfonated compound of this type, fluorene-2,7-di-(sulfonamido-$S^{35}$-2′-naphthalene-X-sulfonic acid) shows a greater localization in tumor tissue at 32 hours than in any other tissue studied (Argus and Hewson, 1954). Thus, the introduction of sulfonic acid groups into the molecule seems to enhance tumor affinity. The possibility that sulfonation might increase the activity of compound I will be studied in a later phase of these investigations.

In Table III is summarized the distribution of radioactivity following administration of the two disulfonamido derivatives of biphenyl. The compound having two free carboxy groups, biphenyl-4,4′-di-(sulfonamidobenzene-4-carboxylic acid)-$S^{35}$ (II), localizes in tumor tissue to a greater extent than in blood cells, spleen, and leg muscle at 2 hours. The concentration in the tumor at 8 hours is the same or greater than that in all other tissues, except the gastrointestinal tract. The high level of this compound in the stomach contents, 2 hours following intravenous injection, suggested possible secretion by the stomach wall. To investigate this, four pylorus-ligated mice were injected intravenously with sulfur 35-labeled compound. For two animals sacrificed at 2 hours, the average recovery of radioactivity from the stomach contents is 0·75 per cent of the administered dose. At 8 hours the average recovery is 2·13 per cent. This secretion of a highly acid compound is not in agreement with the results of Ingraham and Visscher (1935) on the gastric secretion of dyestuffs. These authors found that only basic dyes are secreted by the gastric glands of the dog. Previous work in this laboratory, however, has indicated that an acid dye can be secreted by the rat stomach (Cambel, Breidenbach and Ray, 1954).

Biphenyl-4,4′-di-(sulfonamidobenzene-$S^{35}$-4-sulfonamide) (III) does not show selective tumor affinity. Thus, all body constituents except the blood plasma,

TABLE III.—*Distribution of Radioactivity in Mice Following Intravenous Injection of Disulfonamido Derivatives of Biphenyl*

| | Concentration in μg. compound per g. tissue, or ml. blood cells, or plasma* | | | | Percentage administered radioactivity recovered* | | | |
|---|---|---|---|---|---|---|---|---|
| | Compound II† | | Compound III‡ | | Compound II† | | Compound III‡ | |
| | 2 hr. | 8 hr. | 2 hr. | 8 hr. | 2 hr. | 8 hr. | 2 hr. | 8 hr. |
| Tumor-bearing animals : | | | | | | | | |
| Blood cells | <10·0 | <10·0 | 94·0 | 33·0 | <0·001 | <0·001 | 1·44 | 0·44 |
| Blood plasma | 38·5 | 10·0 | 268·0 | 20·0 | 0·84 | 0·23 | 6·16 | 0·42 |
| Liver | 82·5 | 17·5 | 450·0 | 82·0 | 2·44 | 0·48 | 14·34 | 2·25 |
| Lungs | 41·5 | 13·0 | 254.0 | 72·0 | 0·08 | 0·03 | 0·67 | 0·19 |
| Spleen | 9·0 | 3·0 | 62·0 | 15·0 | 0·04 | 0·01 | 0·28 | 0·06 |
| Kidney | 42·5 | 9·0 | 644·0 | 76·0 | 0·30 | 0·06 | 4·98 | 0·50 |
| Skin | 102·5 | 10·0 | 238·0 | 28·0 | 0·22 | 0·03 | 0·68 | 0·05 |
| Leg muscle | 14·0 | 0·0 | 58·0 | 10·0 | 0·04 | 0·00 | 0·24 | 0·04 |
| Stomach plus contents | 308·0 | 41·5 | 155·0 | 296·0 | 1·54 | 0·18 | 0·92 | 1·20 |
| Small intestine plus contents | 1129·0 | 89·0 | 492·0 | 252·0 | 33·76 | 2·44 | 19·92 | 11·48 |
| Large intestine plus contents | 1405·0 | 2410·0 | 510·0 | 2050·0 | 21·58 | 35·73 | 10·44 | 36·50 |
| Carcass | 36·5 | 10·0 | 95·0 | 18·0 | 14·04 | 3·94 | 37·82 | 5·84 |
| Excreta | — | — | — | — | 15·22 | 35·57 | 6·40 | 28·40 |
| Tumor | 30·0 | 17·5 | 60·0 | 25·0 | 0·49 | 0·38 | 0·41 | 0·16 |
| Total | — | — | — | — | 90·59 | 79·08 | 104·70 | 87·53 |
| Tumor-free animals : | | | | | | | | |
| Blood cells | <10·0 | <10·0 | 112·0 | 40·0 | <0·001 | <0·001 | 1·55 | 0·45 |
| Blood plasma | 39·5 | 10·5 | 370·0 | 106·0 | 0·84 | 0·24 | 7·79 | 1·86 |
| Liver | 97·5 | 12·5 | 402·0 | 142·0 | 2·56 | 0·34 | 11·44 | 3·27 |
| Lungs | 85·5 | 46·5 | 411·0 | 74·0 | 0·22 | 0·14 | 1·33 | 0·18 |
| Spleen | 10·0 | 4·0 | 128·0 | 35·0 | 0·02 | 0·01 | 0·28 | 0·04 |
| Kidney | 46·5 | 8·0 | 1022·0 | 572·0 | 0·36 | 0·06 | 7·06 | 4·01 |
| Skin | 110·0 | 12·5 | 298·0 | 100·0 | 0·24 | 0·03 | 0·80 | 0·16 |
| Leg muscle | 16·5 | 0·0 | 88·0 | 22·0 | 0·06 | 0·00 | 0·34 | 0·08 |
| Stomach plus contents | 274·0 | 54·0 | 142·0 | 78·0 | 1·27 | 0·22 | 1·06 | 0·33 |
| Small intestine plus contents | 844·0 | 114·0 | 361·0 | 345·0 | 26·60 | 3·16 | 16·24 | 13·96 |
| Large intestine plus contents | 1000·0 | 2085·0 | 235·0 | 1734·0 | 20·94 | 41·22 | 5·04 | 32·48 |
| Carcass | 28·0 | 11·5 | 128·0 | 42·0 | 11·18 | 4·80 | 46·60 | 13·18 |
| Excreta | — | — | — | — | 12·41 | 23·45 | 5·18 | 29·62 |
| Total | — | — | — | — | 76·70 | 73·67 | 104·71 | 99·62 |

* Each value is the average from 4 animals.
† Biphenyl-4,4′-di-(sulfonamidobenzene-4-carboxylic acid)-$S^{35}$.
‡ Biphenyl-4,4′-di-(sulfonamifobenzene-$S^{35}$-4-sulfonamide).

spleen, leg muscle and carcass concentrate more radioactive material at 8 hours than does the tumor (Table III). In previous studies, another compound that failed to localize in tumor tissue, fluorene-2,7-di-(sulfonamido-2′-naphthalene)-$S^{35}$, did concentrate in the liver and spleen of tumor-free $CAF_1$/Jax mice to a greater extent than in these organs of mice bearing a transplanted squamous cell tumor (Argus and Hewson, 1954). The possible value of this phenomenon in cancer diagnosis led to more extensive studies which showed that difference in uptake of this compound exists also in other strains and species of animals, and with various types of transplanted tumors (Argus, Hewson and Ray, 1956 ; Argus, Lemasters, Gutierrez and Ray, 1957 ; and Argus, Seepe, Kane and Ray, 1958). Since biphenyl-4,4′-di-(sulfonamidobenzene-$S^{35}$-4-sulfonamide) (III) did not show selective tumor localization, differences in the uptake of this compound by the tissues of tumor-free and tumor-bearing mice were investigated.

The ratios of the concentration of radioactivity in the liver, spleen, kidneys and blood plasma of tumor-free mice to the concentration in these tissues of tumor-bearing mice following administration of biphenyl-4,4′-di-(sulfonamidobenzene-$S^{35}$-4-sulfonamide) (III) are given in Table IV. The liver of tumor-free mice

TABLE IV.—*Ratios of Concentration of Radioactivity in the Tissues of Tumor-free Mice to the Concentration in Tissues of Tumor-Bearing Mice at* 2 *and* 8 *hours following Intravenous Injection of Biphenyl*-4,4′-*di*-(*sulfonamidobenzene*-$S^{35}$-4-*sulfonamide*)

| | Ratio* | |
|---|---|---|
| | 2hr. | 8 hr. |
| Liver | 0·89 | 1·73 |
| Spleen | 2·06 | 2·33 |
| Kidneys | 1·59 | 7·53 |
| Blood plasma | 1·38 | 5·30 |

$$*\left(\frac{\text{Concentration in tissue of tumor-free mice}}{\text{Concentration in tissue of tumor-bearing mice}}\right);$$

Concentration is expressed as $\mu$g. compound/g. of tissue or ml. blood plasma ; each concentration value is the average from 4 animals.

localizes this compound in greater concentration than does the liver of tumor-bearing mice at 8 hours, but not at 2 hours. The phenomenon is present for the spleen at both time intervals studied. At 8 hours this ratio for the liver is about the same as for fluorene-2,7-di-(sulfonamido-2′-naphthalene)-$S^{35}$ in $CAF_1$/Jax mice ; the ratio of the spleen is, however, less than that for the fluorene derivative (Argus, Hewson and Ray, 1956).

With biphenyl-4,4′-di-(sulfonamidobenzene-$S^{35}$-4-sulfonamide) (III) there are two features not found with previous compounds. These are the large differences in localization in the kidneys and in the blood plasma of tumor-free and tumor-bearing animals. Thus, at 8 hours the kidneys of control animals localize 7·53 times as much radioactive material as do the kidneys of tumor-bearing animals (Table IV). This difference in the level of compound in the kidneys is not reflected in the rate of excretion (Table III), since the percentage of administered dose recovered from the excreta is approximately equal for tumor-free and tumor-bearing animals at 2 and at 8 hours.

Two hours after intravenous injection of biphenyl-4,4′-di-(sulfonamidobenzene-$S^{35}$-4-sulfonamide) (III) the concentration of radioactivity in the blood plasma of tumor-free mice is 1·38 times that in the plasma of tumor-bearing mice. At 8 hours the ratio increases to 5·30. Since the difference is measurable in a readily accessible body fluid, this phenomenon may have greater promise as a diagnostic tool. Further studies were therefore carried out at different times following tumor transplantion and tumor excision. The ratios obtained for the blood plasma 2 hours after administration of the biphenyl compound are shown in Table V. Four

TABLE V.—*Concentration of Biphenyl-4,4′-di-(sulfonamidobenzene-$S^{35}$-4-sulfonamide) in the Blood Plasma of Tumor-free, Tumor-bearing, and Tumour-excised $CAF_1$/Jax Mice 2 hours after Intravenous Injection*

| Mice | Concentration* | Ratio† |
|---|---|---|
| Tumor-free | 370‡ | 1·00 |
| Bearing tumor 4 days | 354§ | 1·05 |
| Bearing tumor 3 weeks | 290§ | 1·28 |
| Bearing tumor 4 weeks | 268‡ | 1·38 |
| Three days after tumor excision¶ | 231§ | 1·60 |
| Three weeks after tumor excision¶ | 330§ | 1·12 |

* μg. compound/ml. plasma.
† $\left(\frac{\text{Concentration in tumor-free plasma}}{\text{Concentration in tumor-bearing plasma}}\right)$.
‡ Average value from 4 animals.
§ Average value from 2 animals.
¶ Tumor excised 4 weeks after transplantation.

days after transplantation a slight increase in the ratio is noted ; at 3 weeks the ratio increases to 1·28 ; at 4 weeks the ratio is 1·38. Tumor excisions were made at 4 weeks following transplantation ; no metastases had occurred prior to excision of the tumor. The ratio is still high (1·60) 3 days after excision, indicating that the animals have not yet begun to return to normal ; by 3 weeks the ratio drops to 1·12. These ratios indicate that the capacity of blood plasma to concentrate this biphenyl disulfonamide is influenced by the presence or absence of a tumor in the animal body.

In conclusion, previous and present studies on this subject indicate that at least two factors are important for tumor localization : (*a*) an optimum size of the molecule, and (*b*) the presence of strong acidic groups. Thus, of fluorene-2, 7-disulfonamide-$S^{35}$, fluorene-2,7-di-(sulfonamidobenzene)-$S^{35}$, and fluorene-2,7-di-(sulfonamido-2′-naphthalene)-$S^{35}$, only fluorene-2,7-di-(sulfonamidobenzene)-$S^{35}$, the compound of intermediate size, shows selective tumor localization. However, by introducing sulfonic acid groups into the naphthalene moiety of fluorene-2,7-di-(sulfonamido-2′-naphthalene)-$S^{35}$, the compound acquires tumor affinity, Similarly fluorene-2,7-disulfonic acid-$S^{35}$ shows selective localization. This enhancing ability of acidic groups in the molecule is also evident in comparing the tumor localization of the two derivatives of biphenyl investigated here. Thus, biphenyl-4,4′-di-(sulfonamidobenzene-4-carboxylic acid)-$S^{35}$ shows higher tumor localization ratios than biphenyl-4,4′-di-(sulfonamidobenzene-$S^{35}$-4-sulfonamide).

While studies with fluorene-2,7-di-(sulfonamido-2′-naphthalene)-$S^{35}$ have shown that the presence of a tumor in the animal body affects the ability of the spleen and liver to localize the compound, the present work with biphenyl-4,4′-di-(sulfonamidobenzene-$S^{35}$-4-sulfonamide) suggests that the presence of a neoplasm also may influence the ability of the blood and kidneys to concentrate certain large molecular weight compounds. What seems to be of importance, from the diagnostic standpoint, is that a difference in the localizing ability of a tissue in tumor-free and tumor-bearing animals has been demonstrated in a readily accessible body constituent.

### SUMMARY

1. Syntheses are described for fluorene-2,7-di-(sulfonamidobenzene)-$S^{35}$, biphenyl-4,4′-disulfonyl chloride-$S^{35}$, biphenyl-4,4′-di-(sulfonamidobenzene-4-

carboxylic acid)-$S^{35}$ and biphenyl-4,4′-di-(sulfonamidobenzene-$S^{35}$-4-sulfonamide).

2. The distribution of radioactivity in tumor-bearing and tumor-free mice following intravenous injection of the three disulfonamides showed fluorene-2,7-di-(sulfonamidobenzene)-$S^{35}$ to have the most favorable tumor localization.

3. Experiments with biphenyl-4,4′-di-(sulfonamidobenzene-4-carboxylic acid)-$S^{35}$ in pylorus-ligated mice revealed this acid compound to be secreted by the gastric mucosa.

4. Biphenyl-4,4′-di-(sulfonamidobenzene-$S^{35}$-4-sulfonamide) is concentrated by the liver, spleen, kidneys and blood plasma of tumor-free mice to a greater extent than by these organs of tumor-bearing mice, thus revealing diagnostic possibilities.

This work was supported by reasearch grant C-1356 from the National Cancer Institute of the National Institutes of Health, U.S. Public Health Service.

### REFERENCES

Argus, M. F.—(1953) *Brit. J. Cancer*, **7**, 273.
*Idem* and Hewson, K.—(1954) *Ibid.*, **8**, 698.
*Idem*, Hewson, K. and Ray, F. E.—(1956) *Ibid.*, **10**, 321.
*Idem*, Lemasters, T. J., Gutierrez, N. and Ray, F. E.—(1957) *Proc. Amer. Ass. Cancer Res.*, (3), **2**, 185.
*Idem*, Seepe, T. L., Kane, J. F. and Ray, F. E.—(1958) *Ibid.*, (4), **2**, 277.
Cambel, P., Breidenbach, A. W. and Ray, F. E.—(1954) *Amer. J. Physiol.*, **178**, 493.
Ingraham, R. C. and Visscher, M. B.—(1935) *J. gen. Physiol.*, **18**, 695.
Klein, M., Argus, M. F. and Ray, F. E.—(1953) *Brit. J. Cancer*, **7**, 264.
Oakley, C. L. and Warrack, G. H.—(1940) *J. Path. Bact.*, **50**, 372.